# *Bacillus thuringiensis*-derived Cry5B Has Potent Anthelmintic Activity against *Ascaris suum*

**DOI:** 10.1371/journal.pntd.0002263

**Published:** 2013-06-20

**Authors:** Joseph F. Urban, Yan Hu, Melanie M. Miller, Ulrike Scheib, Ying Y. Yiu, Raffi V. Aroian

**Affiliations:** 1 USDA, Agricultural Research Service, Beltsville Human Nutrition Research Center, Diet, Genomics, and Immunology Laboratory, Beltsville, Maryland, United States of America; 2 Section of Cell and Developmental Biology, Division of Biological Sciences, University of California, San Diego, La Jolla, California, United States of America; McGill University, Canada

## Abstract

*Ascaris suum* and *Ascaris lumbricoides* are two closely related geo-helminth parasites that ubiquitously infect pigs and humans, respectively. *Ascaris suum* infection in pigs is considered a good model for *A. lumbricoides* infection in humans because of a similar biology and tissue migration to the intestines. *Ascaris lumbricoides* infections in children are associated with malnutrition, growth and cognitive stunting, immune defects, and, in extreme cases, life-threatening blockage of the digestive tract and aberrant migration into the bile duct and peritoneum. Similar effects can be seen with *A. suum* infections in pigs related to poor feed efficiency and performance. New strategies to control *Ascaris* infections are needed largely due to reduced treatment efficacies of current anthelmintics in the field, the threat of resistance development, and the general lack of new drug development for intestinal soil-transmitted helminths for humans and animals. Here we demonstrate for the first time that *A. suum* expresses the receptors for *Bacillus thuringiensis* crystal protein and novel anthelmintic Cry5B, which has been previously shown to intoxicate hookworms and which belongs to a class of proteins considered non-toxic to vertebrates. Cry5B is able to intoxicate *A. suum* larvae and adults and triggers the activation of the p38 mitogen-activated protein kinase pathway similar to that observed with other nematodes. Most importantly, two moderate doses of 20 mg/kg body weight (143 nM/kg) of Cry5B resulted in a near complete cure of intestinal *A. suum* infections in pigs. Taken together, these results demonstrate the excellent potential of Cry5B to treat *Ascaris* infections in pigs and in humans and for Cry5B to work effectively in the human gastrointestinal tract.

## Author Summary

*Ascaris suum* is an intestinal parasitic nematode of pigs that is very closely related to *Ascaris lumbricoides*, a major intestinal parasitic nematode of humans that infects more than one billion people worldwide. Because of reduced efficacy and the threat of resistance to the current small set of approved drugs to treat *Ascaris* infections, new treatments are needed. Here we test against *A. suum* infections the effectiveness of Cry5B, a nematode-killing protein made by the natural soil bacterium *Bacillus thuringiensis* and representing a promising new class of anthelmintics. We demonstrate for the first time that *A. suum* possesses the receptors to bind Cry5B and that Cry5B can kill *A. suum* larvae and adults in culture. Most importantly, we demonstrate that oral administration of Cry5B to pigs infected with *A. suum* larvae results in a near complete elimination of the infection. Given the similarities between *A. suum* and *A. lumbricoides* and the similarity between the pig and human gastrointestinal tracts, our data indicate that Cry5B has excellent potential to treat *Ascaris* infections in veterinary animals and in humans.

## Introduction

*Ascaris lumbricoides*, the large roundworm, is the most common parasitic nematode infection of humans with estimates of more than one billion people infected worldwide in mostly lesser developed countries [1]. Chronic infections in humans are associated with growth and cognitive stunting, impaired nutritional status, and dysfunctional immune regulation related to a polarized Th2 immunity and altered protective antibody responses to bacterial vaccination [1], [2], [3], [4]. More acute symptoms associated with larval migration through lung tissue involve inflammation, breathing difficulties, and fever [4], [5]. Adult *Ascaris* infections can lead to abdominal pain, nausea and diarrhea, and potentially life-threatening blockage of the intestinal or biliary tracts [4], [6]. Although treatable with approved anthelmintics, the arsenal of available drugs against nematodes is largely limited to classes of benzimidazoles, macrocyclic lactones, and imidazothiazoles, and *Ascaris* drug resistance in human therapy is a rising concern [7], [8], [9], [10].

*Ascaris suum* is a common infection in pigs and a significant and costly management problem in confinement and free-range facilities because of high worm fecundity and resistance of infective eggs to environmental stressors [11]. Adverse effects in pigs are similar to those in humans and include migrating larvae-induced liver scaring (white spots), pulmonary inflammation, reduced nutritional absorption and feed efficiency, altered responses to bacterial vaccination, and a polarized Th2 immunity [4], [12], [13], [14], [15]. *Ascaris suum* and *A. lumbricoides* are genetically similar and there is currently some evidence that they are the same species [16].

Crystal (Cry) proteins made by the soil-bacterium *Bacillus thuringiensis* (Bt) are vertebrate-safe proteins used extensively and successfully on organic and conventional agriculture, in transgenic food and non-food crops, and in vector-control programs to kill insect pests [17], [18], [19]. Cry proteins act as ingested pore-forming proteins that target invertebrate-restricted intestinal receptors [20]. The Cry protein Cry5B has been shown to be effective against two intestinal parasitic nematode infections in rodents when administered orally via gavage [21], [22], [23]. Specifically, a single dose of 100 mg/kg body weight (BW) of Cry5B is able to eliminate 90% of *Ancylostoma ceylanicum* hookworm parasites from infected hamsters (*A. ceylanicum* is a zoonotic hookworm species capable of infecting humans and causing hookworm disease [24]) and eliminate 70% of *Heligmosomoides bakeri* (previously designated as *H. polygyrus*) parasites from the intestine of mice (*H. bakeri* is a murine-specific parasitic nematode [25]). The receptors for Cry5B in nematodes are invertebrate-specific glycolipids present on the nematode intestinal mucosa [21], [23], [26], [27], [28], [29], [30]. Both *H. bakeri* and *A. ceylanicum* are phylogenetically related Clade V nematodes [31]. Here we test for the first time if *A. suum*, a Clade III nematode [31], possesses Cry5B-binding receptors and if Cry5B is able to intoxicate *A. suum in vitro*. We further test Cry5B therapeutic efficacy *in vivo* against an experimental *A. suum* infection in pigs, a comparable *in vivo* model for both *A. lumbricoides* infections in humans and for the efficacy of Cry5B protein in the human gastrointestinal (GI) tract.

## Materials and Methods

### IACUC/Ethics committee

All animal experiment was carried out under protocols approved by the Beltsville Area Animal Care and Use Committee (BAACUC), protocol number 10-012. All housing and care of laboratory animals used in this study conform to the NIH Guide for the Care and Use of Laboratory Animals in Research (see 18-F22) and all requirements and all regulations issued by the USDA, including regulations implementing the Animal Welfare Act (P.L. 89-544) as amended (see 18-F23).

### *Ascaris* lifecycle and parasite preparations

Adult *A. suum* female worms were obtained from naturally infected sows collected at the Beltsville Agricultural Research Center Abattoir. The proximal 2 cm of uteri from each female was dissected and aseptically transferred to 100 mm culture dishes where the pooled uteri were macerated with a syringe plunger and the mixture passed through a sterile 100 um nylon sieve (BD Falcon #352360, Bedford, MA) to provide a single suspension of eggs. The egg suspension was washed with PBS containing 200 µg/ml penicillin, 200 units/ml streptomycin and 10 µg/ml amphotericin B (antibiotics) three times by centrifugation at 200×g after removal of intervening supernatant washes by suction. The final suspension was placed into 100 mm culture dishes and maintained at ambient room temperature for at least 60 days (with occasional washing of the eggs) to embryonate the eggs to an infective stage. The suspension was counted for eggs with well-formed and ensheathed larvae and stored at 4°C until used.

Experimental pigs were obtained from a facility at the Beltsville Agricultural Research Center, Beltsville, MD. Pigs were derived from boars from a four-way crossbred composite BX line (Duroc X maternal Landrace X terminal Landrace X Yorkshire) designed by scientists at the USDA/ARS/US Meat Animal Research Center, Clay Center, NE to be genetically similar to genetics in the commercial swine industry at the time they were born; the genetics of the gilts are predominantly of the BX composite line. Pigs were from a herd screened yearly for porcine reproductive and respiratory syndrome virus (PRRSV), influenza (H1N1 and H3N2), pseudorabies, and brucellosis by the Veterinary Services Group at the Beltsville Agricultural Research Center and have been negative for these infections. They were individually housed in stalls with a non-absorptive concrete floor surface with *ad libitum* access to water and fed a nutritionally adequate corn/soybean-based feed once per day.

Pigs used to collect larval and adult stages of *A. suum* were at least eight weeks of age when inoculated *per os* with a suspension of approximately 10,000 to 30,000 infective eggs. To collect the parasitic and adult stages of *A. suum* for in vitro testing with Cry5B, infected pigs were euthanized using Euthasol (Virbac AH, Inc., Fort Worth, TX) with doses described by the manufacturer at various times after inoculation and the entire small intestine was removed. The intestinal tract was opened with a scissor and the contents and mucosa rubbed between two figures into a large vessel over a slow stream of warm tap water. Larvae were isolated by an agar-gel technique [32]. Briefly, the intestinal suspension was mixed with an equal volume of molten 2% agar and poured into pans containing a cheese cloth to form a solid gel which was then placed into containers of 0.85% NaCl (saline) warmed to 37°C and incubated for 2 hrs. After removal of the gel from the holding container, larvae were isolated by decanting the fluid and washing the larvae with several volumes of warm saline and settling in conical glasses for several repetitions. The larval suspension was then transferred to sterile 50 ml culture tubes and aseptically washed (repeated settling of larvae and decanting of supernatants) with warm RPMI1640 media containing antibiotics five times followed by a one hr incubation at 37°C and then five additional washes. The larval suspensions were shipped overnight from Beltsville, MD to La Jolla, CA in sealed 50 ml tubes with media containing 10% FBS. Adult worms were collected from intestinal contents passed over copper separation screens, washed and shipped similar to the larval stages.

### Glycolipid binding, p38 phosphorylation, and *in vitro* intoxication experiments

For glycolipid experiments, *Caenorhabditis elegans* was grown and prepared as described [30]. Early fourth-stage *A. suum* larvae (L4) collected from the intestine at 14 days post-inoculation (PI) were washed in water and resuspended in three pellet volumes of water. The *A. suum* pellet was shock frozen with liquid nitrogen in a porcelain mortar, and the frozen pellet was ground with a pestle until it thawed. After repeating the freezing and grinding steps four times, the suspension was sonicated four times. Complete membrane disruption was verified by microscopy. Upper phase glycolipids were purified based on the Svennerholm partitioning method [33], except that upper phase *A. suum* glycolipids were applied on a tC18 silica cartridge (Millipore) four times (instead of two). The thin-layer chromatography (TLC) assay was carried out as previously described in [30]. Briefly, purified upper phase glycolipids were separated on a HPTLC plates in a chamber filled with 4∶4∶1 chloroform∶methanol∶water. Developed plates were either stained with a non-specific orcinol stain to ensure comparable glycolipid loading of different nematodes or probed with biotinylated Cry5B [29]. The TLC Cry5B competition assays were undertaken in the presence of 100 mM glucose or galactose. Cry5B binding was visualized with the help of the avidin/alkaline phosphatase (Reagent A/B) Vectastain ABC-AP kit. The experiment was performed three times, with a representative trial shown here.

For phospho-p38 experiments, day 14 L4 were exposed to either no protein or 100 µg/mL Cry5B (10 larvae per condition) for 2 hr (37°C, 5% CO_2_) in RPMI 1640 plus 5% fetal bovine serum plus antibiotics (100 U/mL penicillin, 100 µg/mL streptomycin; 0.25 µg/mL fungazole). Harvesting and processing of larvae and Western blotting were carried out as described [23]. The experiment was independently performed a total of four times with similar results; one representative result is shown. The intensities of the bands were quantitated using NIH Image J (v1.41). The intensities of the α-tubulin bands were very similar and were used to normalize the intensities of the phospho-p38 bands.

The medium used for *in vitro* assays was the same as for p38 phosphorylation experiments. *In vitro* assays with L4s were carried out in a 500 µl volume in a 24-well plate. There were 5–6 L4 of mixed genders per well because of the difficulty to discern larval gender at this stage of development. In each independent trial, there were two wells/dose (10–12 L4s total). For day 14 PI larvae, three independent trials were performed; for day 19 PI larvae, two independent trials were performed. For control wells, 10 µL 20 mM Hepes pH 8.0 buffer was added in place of Cry5B (always added as 10 µL in 20 mM Hepes pH 8.0; final concentrations = 0.1 µg/mL, 1.0 µg/mL, 10.0 µg/mL, 100.0 µg/mL, and 1000 µg/mL). For day 14 PI larvae, the effective dose 50% (ED_50_) value was calculated at day 4 (half way through the experiment) and day 7 (completion of experiment) using PROBIT [34]. For day 19 PI larvae, we did not calculate ED_50_ values as all the doses, by and large, behaved similarly.

Adult worm assays were carried out in 125 mL or 250 mL flasks (buffer volume 10 mL or 20 mL respectively) with one worm/flask (2 worms/dose/trial with two independent trials). For control flasks, 20 mM Hepes was added in place of Cry5B (one dose, 100 µg/mL). The medium in the flasks was refreshed/changed every two days. Plates or flasks were put at 37°C in a 5% CO_2_ in air incubator. The assays were scored daily over the period of one week. Larvae that moved in the absence or presence of gentle touch with an eyelash were scored as alive; larvae that did not move were scored as dead. A motility index score on a scale from 3-to-0 was used to determine adult worm viability: 3 represents a parasite with vigorous movement similar to control no drug at the start of the experiment; 2 represents a parasite with whole-body movements (seen without external stimulus) significantly slower than control no drug at the start; 1 represents a parasite that was not moving on its own but moved when prodded with a wooden inoculating stick (tested at three different body locations); and 0 represents a worm that did not move even when prodded.

### *In vivo* experiment

To determine the *in vivo* efficacy of Cry5B against *A. suum*, a total of ten recently weaned four week old pigs from two different litters were split into two groups of five pigs each. All ten pigs were inoculated per os with 5,000 infective *A. suum* eggs and at ten and 12 days PI five pigs (placebo group) were given *per os* a suspension of spore lysate (HD1 parent strain) and five pigs (Cry5B-treated group) were given spore crystal lysate (HD1 parent strain with Cry5B-expressing vector) at a final dose of 20 mg/kg BW of Cry5B. HD1 Bt lysates with and without Cry5B were prepared as described [22]. Spore counts were checked and were found similar for both groups. Pigs were euthanized on day 15 PI with infective eggs and L4 isolated from the intestinal contents by the gel-agar method. The total number of L4 from each pig was counted under a dissecting microscope and average number of L4 recovered from each group were calculated and compared for a statistical difference using Student's t-test assuming unequal variances. The recovered L4 were fixed with 10% buffered-formalin and the length of each larva was subsequently measured microscopically using the Olympus MicroSuite – B3SV system at 40× magnification (Olympus America, Melville, NY). Briefly, aliquots of the larval suspension were transferred to a microscope slide and covered with a glass slip. The slide was scanned and at least 10 larvae from each pig were manually traced using a computer mouse. The length of each larva was calculated in µm using calibrated software and the average ± the standard error of the mean recorded for the two treatment groups.

## Results

### *Ascaris suum* expresses Cry5B receptors

**Figure 1:**
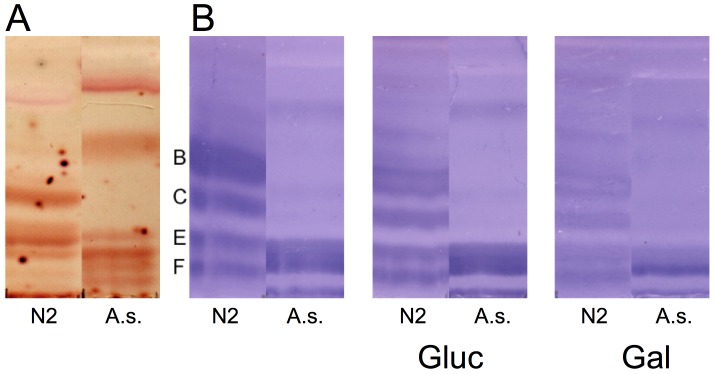
*A. suum* expresses Cry5B glycolipid receptors. **A.** Orcinol staining of upper phase glycolipids from mixed-staged *C. elegans* (N2) and *A. suum* fourth-stage larvae (A.s.). The glycolipid profiles of the two are different. **B.** Cry5B binding to glycolipids from *C. elegans* (N2) and *A. suum* (A.s.) in the absence of sugar (left panel), presence of 100 mM glucose (middle panel), and presence of 100 mM galactose (right panel). The profile of Cry5B-binding glycolipids (left panel) is different from both nematodes (bands B, C, E, and F refer to Cry5B-binding glycolipids as noted in [27]). The intensity of all bands is reduced in the presence of galactose.

To determine if *A. suum* expresses Cry5B-binding glycolipid receptors, glycosphingolipids were isolated from *A. suum* L4 and separated by thin-layer chromatography (Fig. 1A). These glycolipids were then probed with protease-activated, biotinylated Cry5B. *Ascaris suum* L4 contain several Cry5B-binding glycolipids that show a profile somewhat different than that of *C. elegans* (Figure 1B). The specificity of the Cry5B binding was determined by competition experiments. As previously found with *C. elegans*, *A. ceylanicum*, and *P. pacificus* [21], [27], [30], galactose, more than glucose, is able to compete binding of Cry5B to glycolipids from *A. suum* (Figure 1B).

### *Ascaris suum* larvae and adults are susceptible to Cry5B *in vitro*

**Figure 2:**
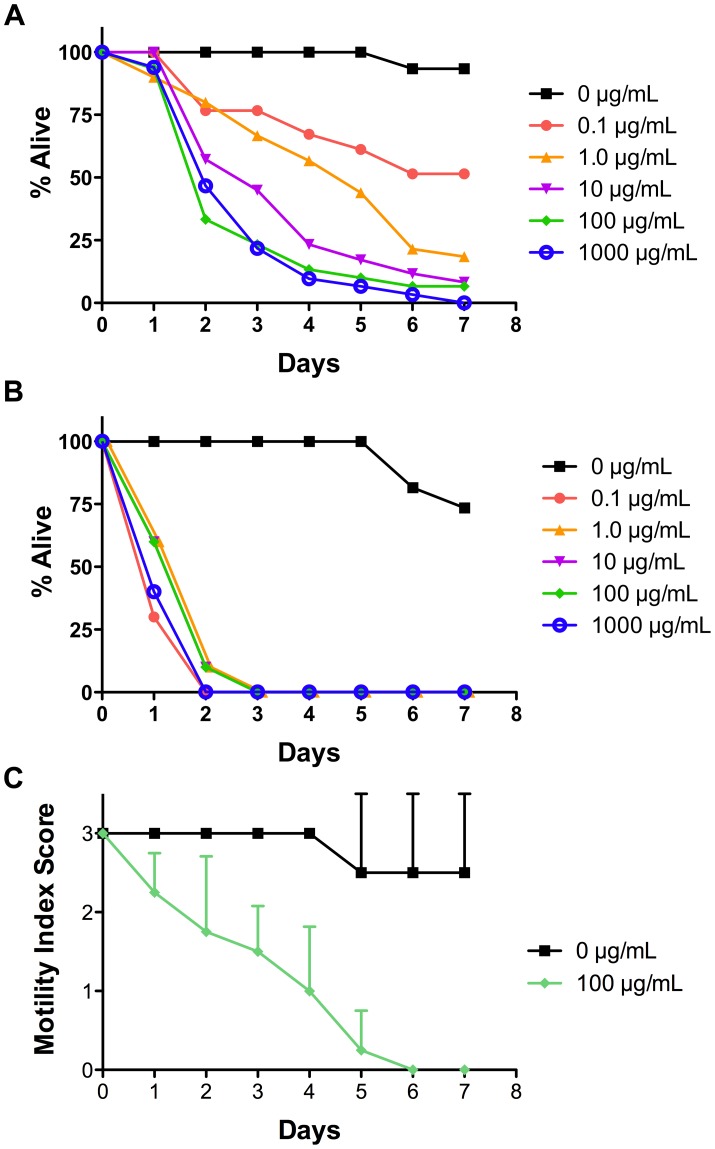
*Ascaris suum* larvae and adults are susceptible to Cry5B *in vitro*. **A.** The % live (motile) day 14 post-inoculation (PI) fourth-stage larvae (L4) scored on a daily basis at various doses of Cry5B (three trials combined as one); n = 30–36/condition. Legend indicates the dose of Cry5B. **B.** The % live (motile) day 19 PI L4 scored on a daily basis at various doses of Cry5B (two trials combined as one); n = 20–24/condition **C.** Average motility index score of adults exposed to 100 µg/mL Cry5B (days 54 and 89 PI combined as one) scored on a daily basis. Motility index is as defined in Methods. Error bars indicate standard deviation. n = 4/condition.

After determining that *A. suum* expresses Cry5B receptors, we asked whether parasite viability is susceptible to Cry5B *in vitro*. Larvae and adults were isolated from the pig small intestine at 14 days PI (early stage L4), 19 days PI (mid stage L4), and 54 or 89 days PI (adult stage) [35]. Worms were incubated in culture media with various doses of Cry5B and scored daily for intoxication based on motility after gentle physical touching. Conditions were repeated either twice (day 19 PI L4 or adults) or three times (day 14 PI L4). At all parasitic stages, measurable intoxication was seen with time in culture (Figure 2A, B, C). Day 14 L4s displayed a nice dose-dependent response to Cry5B. For day 14 L4s, we calculated an ED_50_ value at day 4 of 1.1 µg/mL (95% confidence interval 0.30–3.1) and at day 7 of 0.094 µg/mL (95% confidence interval 0.011–0.30). Day 19 L4s were even more sensitive to Cry5B, with quicker intoxication even at the lowest doses used.

Adult parasites exposed to Cry5B were largely immotile by day 4 (index score 1), while controls were still vibrant (index score 3). The difference is most striking by day 6. On day 6, control adult parasites were mostly very healthy – three out of four were fully motile, all scoring as a 3, and one out of four moved only when touched, scoring as a 1. Conversely, on day 6 all four of the adult parasites in Cry5B were apparently dead (did not move even when touched multiple times). The two groups are statistically different on day 6 (P = 0.018, 2-sample Kruskal-Wallis Test). The data demonstrate that *A. suum* is biologically susceptible to Cry5B as L4 and adult stages from the intestine.

### *Ascaris suum* larvae activate p38 in response to Cry5B

**Figure 3:**
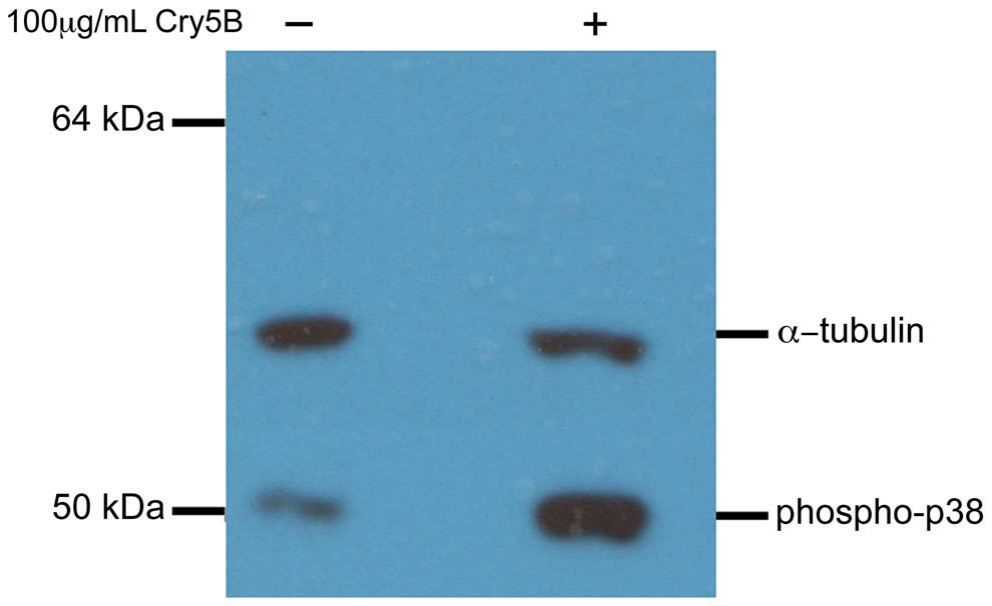
Cry5B treatment activates p38 MAPK in *A. suum* fourth-stage larvae (L4). *Ascaris suum* L4 were exposed to either buffer or Cry5B and then protein extracts prepared. Treatment with Cry5B results in significant up-regulation of activated (phosphorylated) p38 MAPK. α-tubulin levels are used to normalize total protein loading.

A molecular hallmark of nematode intoxication by Cry5B is activation of the p38 mitogen-activated protein kinase (MAPK) pathway upon exposure to the pore-forming protein [23], [36], [37]. To ascertain whether intoxication of *A. suum* by Cry5B results in a similar response, *A. suum* L4 were exposed to Cry5B and probed for phosphorylated (activated) p38 levels by immunoblotting. As before, total protein levels were controlled using an α-tubulin antibody [23], [36], [37]. As shown in Figure 3, treatment of *A. suum* with Cry5B results in significant up-regulation of phospho-p38. Densitometric analyses indicate that this up-regulation is 4.3 fold.

### Cry5B provides significant chemotherapy against *A. suum in vivo*

**Figure 4:**
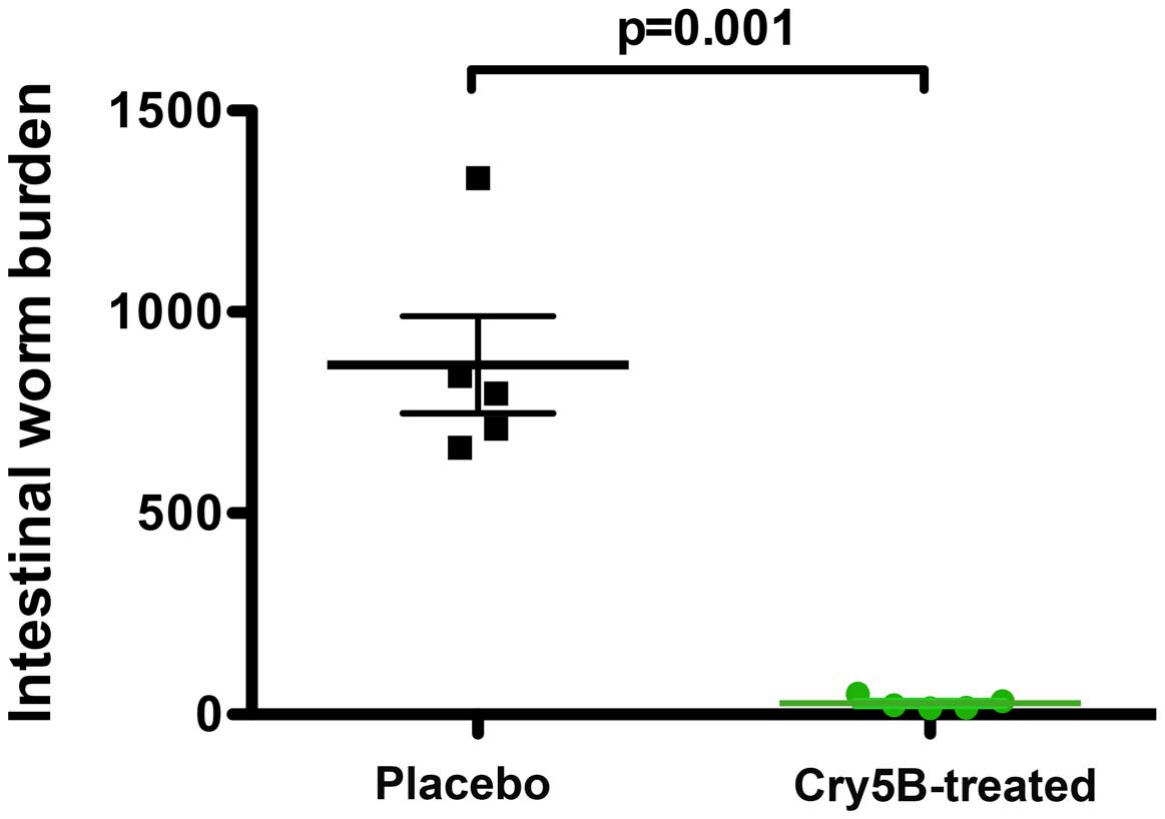
Cry5B effects a near complete cure of *Ascaris suum* infection in pigs. Shown are the fourth-stage larval burdens of individual pigs treated with HD1 spore lysate (placebo; parent strain without Cry5B-expressing plasmid) and HD1+Cry5B spore-crystal lysate (Cry5B-treated; parent strain with Cry5B-expressing plasmid). The actual worm burden means with standard error of the means are, respectively, 868.6±120 and 27.6±6.4.

The therapeutic efficacy of Cry5B *in vivo* was tested in pigs infected with *A. suum*. Ten pigs were each inoculated with approximately 5,000 infective *A. suum* eggs. Five infected pigs (Cry5B-treated group) received two 20 mg/kg BW (143 nM/kg BW) doses of Cry5B via gavage, one dose each on days 10 and 12 PI (Cry5B was administered as a spore-crystal lysate; [22]); the other five infected pigs (placebo group) received one dose of a spore lysate without Cry5B on those same days. On day 15 PI, the pigs were euthanized and worm burdens (L4) in the small intestine were assessed. The two-dose Cry5B treatment resulted in a near complete (97%) elimination of *A. suum* L4 (Figure 4). All pigs appeared clinically normal after egg inoculation and spore lysate treatments. In addition, microscopic tracings of the length of individual *A. suum* L4 recovered from Cry5B-treated pigs averaged 3937±86 µm and was significantly different from an average length of 5796±205 µm from the placebo-treated pigs, suggesting that the growth of the few L4 remaining in the intestine was severely affected by Cry5B. These results indicate Cry5B has great potential to treat *Ascaris* infections in humans.

## Discussion

Here we demonstrated for the first time that a *Bacillus thuringiensis* crystal protein (Cry5B) can intoxicate *Ascaris suum* parasites *in vitro* and can reduce growth and facilitate expulsion of parasites from the intestine of pigs *in vivo*. Intoxication *in vitro* was observed for the fourth-stage larvae (L4) and adults isolated from the pig intestine. Consistent with its ability to intoxicate *A. suum*, Cry5B glycolipid receptors were isolated from *A. suum* larvae. Furthermore, as seen previously with *C. elegans*, *A. ceylanicum*, and *P. pacificus*, receptor binding was inhibited by galactose competition. Similar to results with *C. elegans* and *A. ceylanicum*, treatment with Cry5B also results in significant up-regulation of phospho-p38 MAPK. Taken together, these data suggest that Cry5B intoxicates *A. suum* in a manner similar to other nematodes.

That *A. suum* expresses as least one Cry5B-binding glycolipid species could be predicted from the literature. The phosphorylcholine substituted glycolipid species in *C. elegans* [Gal(β1-3)Gal(α1-3)GalNAc(β1-4)[PC-6]GlcNAc(β1-3)Man(β1-4)Glc(β1-1)ceramide] that binds Cry5B [27] is perfectly conserved in *A. suum* [38]. This species is apparent as a Cry5B-binding glycolipid “E” in our overlay (Figure 1B; [27]) and is also found in the human parasite *A. lumbricoides* [39].

Strikingly, two 143 nM/kg BW doses of Cry5B were able to nearly completely (97%) eliminate *A. suum* L4 from the intestines of infected pigs (for comparison 143 nM/kg BW of albendazole would equate to 0.04 mg/kg BW). The dose Cry5B used is within the range of other commercially available swine anthelmintics (dichlorvos = 6–9 mg/kg BW; pyrantel pamoate = 22 mg/kg BW; levamisole = 8 mg/kg BW), some also used to eliminate parasitic larval stages of *A. suum* (fenbendazole = 5–10 mg/kg BW for three days) [40]. The timing of Cry5B treatment of pigs was targeted because the early L4 is metabolically similar to the adult [41] and worm accumulation is stable in the intestine between 10 and 17 days after inoculation, a period that precedes a self-cure response by the pig that significantly reduces the number of L4 in the intestine [42]. It is currently impractical to evaluate the efficacy of Cry5B against adult *A. suum in vivo* because of limited current capacities to produce Cry5B in quantities to dose the large number of pigs needed to establish adult worm infections after experimental inoculation and the weight of rapidly growing pigs needed for a minimum of 49 days after inoculation when the worms would become patent and produce eggs. Future studies of the minimum dose and frequency of treatment and the evaluation of tolerability are planned as Cry5B delivery and production systems are optimized and become more cost effective.

This *in vivo* result has several major implications. First, our results importantly show that Cry5B can affect parasitic nematode expulsion in pigs, which possess an intestinal tract functionally similar to that of humans [43], [44]. This finding further sustains the potential of Cry protein anthelmintics for human therapy.

Second, this current study and earlier experiments with infection of hamsters with *A. ceylanicum* [21], [23] demonstrate that Cry5B can significantly bind to and facilitate expulsion from the host of two of the three major intestinal parasitic nematode infections in humans (hookworms and the large roundworm *Ascaris*). This result is important because of the limited arsenal of anthelmintics available to treat human parasitic nematode infection and newer anthelmintics like monepantel, developed to treat gastrointestinal nematode parasites in sheep, have limited efficacy against *Ascaris* [45].

Third, *A. suum* is in a phylogenetic group (Clade III) distinct from the two other intestinal nematodes tested *in vivo* for Cry5B anthelmintic activity to date, *A. ceylanicum* and *H. bakeri* (both Clade V) [31]. It has also been previously shown that Cry5B intoxicates the plant-parasitic nematodes of the genus *Meloidogyne*, which belong to Clade IV [46], [47]. The data presented here are the first known describing susceptibility of a Clade III nematode to a Bt crystal protein and, together with previous results, suggest that Cry5B can broadly impact parasitic nematode infections.

Fourth, our results demonstrated that Cry5B has excellent utility in treating nematodes of veterinary importance, *e.g.*, *A. suum* infection in pigs. Because Cry5B works mechanistically different from other approved anthelmintics [34], Cry5B could be used in places where there is resistance to current anthelmintics. Of particular interest is the observation that that a Bt strain expressing Cry5B intoxicated *Haemonchus contortus* parasites *in vitro* [48]. This is an important because there is world-wide resistance of *H. contortus* to conventional anthelmintics used in sheep and goats and new anthelmintics are needed to treat economically important ruminants [49]. In addition, anthelmintic resistance in the equine ascarid parasite *Parascaris equorum* is a growing concern [50] and the utility of testing for Cry protein efficacy against an important parasitic ascarid of horses is more economically feasible in swine as a physiologically similar non-ruminant host species.

In summary, these data demonstrate the excellent potential of Bt Cry proteins as a major new class of anthelmintics to treat mammals of veterinary importance and, most importantly, for the two billion humans infected with intestinal nematodes.
